# Lactose inhibits regulatory T-cell-mediated suppression of effector T-cell interferon-γ and IL-17 production

**DOI:** 10.1017/S0007114514001998

**Published:** 2014-10-21

**Authors:** Monika Paasela, Kaija-Leena Kolho, Outi Vaarala, Jarno Honkanen

**Affiliations:** 1 Immune Response Unit, Department of Vaccination and Immune Protection, National Institute for Health and Welfare, Haartmaninkatu 8, FI-00290Helsinki, Finland; 2 Children's Hospital, University of Helsinki, Helsinki, Finland

**Keywords:** Lactose, Inflammation, Immunomodulation, T cells

## Abstract

Our interest in lactose as an immunomodulatory molecule results from studies showing that lactose binds to galectin-9, which has been shown to have various regulatory functions in the immune system including regulation of T-cell responses. Impaired regulation of T helper (Th)1 and Th17 type immune responses and dysfunction of regulatory T cells (T_reg_) have been implicated in many human immune-mediated diseases. In the present study, we investigated the effects of lactose on immune regulation using co-cultures of human peripheral blood mononuclear cell (PBMC)-derived T_reg_ and effector T cells (T_eff_) obtained from twenty healthy adults. T_reg_, i.e. CD4^+^CD25^+^CD127^−^, were isolated from PBMC by immunomagnetic separation. The fraction of CD4^+^CD127^−^ cells that was depleted of CD25^+^ cells was used as T_eff_. T_reg_ and T_eff_ at a ratio 1:5 were activated and the effects of lactose on the secretion of interferon-γ (IFN-γ) and IL-17 were analysed using ELISA for protein and quantitative RT-PCR for mRNA. T_reg_ down-regulated the secretion of both IFN-γ (8·8–3·9 ng/ml, *n* 20, *P*= 0·003) and IL-17 (0·83–0·64 ng/ml, *n* 15, *P*= 0·04) in co-cultures, while in the presence of lactose the levels of secreted IFN-γ and IL-17 remained high and no down-regulation was observed (16·4 *v*. 3·99 ng/ml, *n* 20, *P*< 0·0001, and 0·74 *v*. 0·64 ng/ml, *n* 15, *P*= 0·005, respectively). We showed that lactose inhibits human T_reg_-mediated suppression of Th1 and Th17 immune responses *in vitro*.

Lactose, a β-galactoside consisting of galactose and glucose residues, is the main carbohydrate in mammalian breast milk. In the first few months of life, lactose provides energy to infants and supports the growth of lactose-fermenting commensals^(^ ^1^ ^)^. During development, the expression of the enzyme lactase starts to diminish and mammals become lactose intolerant, but the exact mechanisms of this developmental decline in enzyme activity are unclear^(^ ^2^ ^,^ ^3^ ^)^. However, in some populations, lactase expression persists due to genetic mutation, and lactose tolerance is maintained during adult life, allowing the use of lactose-containing dairy products^(^ ^4^ ^)^.

Galectin-9 (Gal-9) belongs to the vast group of mammalian lectins that bind to β-galactosides, such as lactose, with a conserved carbohydrate recognition domain^(^ ^5^ ^,^ ^6^ ^)^. Gal-9 is expressed by various cell types, such as macrophages, T cells and intestinal epithelial cells^(^ ^6^ ^–^ ^9^ ^)^. Gal-9 is widely distributed due to its importance in the host system with complex biological functions including antimicrobial immunity, cell adhesion, anti-allergic functions, regulatory T-cell (T_reg_) differentiation and effector T-cell (T_eff_) apoptosis^(^ ^8^ ^–^ ^13^ ^)^. Gal-9 mediates its effects by two receptors: cell-surface protein disulfide isomerase and T-cell Ig and mucin domain-3 (Tim-3)^(^ ^14^ ^,^ ^15^ ^)^. It has been demonstrated in animal models that the Gal-9/TIM-3 pathway regulates antiviral immune responses in cytotoxic T cells and is crucial for shutting down excessive T helper (Th)1 and Th17 immune responses^(^ ^13^ ^,^ ^15^ ^,^ ^16^ ^)^. Tim-3-mediated regulation of Th1 and Th17 immune responses has been shown in human subjects by Hastings *et al.* ^(^ ^17^ ^)^. In some studies, lactose has been used as a Gal-9 antagonist. Similar to *Gal-9* gene silencing, lactose abrogates Gal-9-mediated immune regulation by limiting its engagement with Tim-3^(^ ^18^ ^)^. This results in increased proliferation of T cells and induction of pro-inflammatory responses with aggravation of clinical outcomes in mouse models of experimental autoimmune encephalitis and arthritis^(^ ^13^ ^,^ ^15^ ^,^ ^16^ ^,^ ^19^ ^)^.

Although proper Th1 and Th17 immune responses are required for host defence in intracellular pathogen clearance and mucosal antimicrobial immunity, respectively, uncontrolled and excessive Th1 and Th17 immune activity may have detrimental effects and may result in the development of immune-mediated diseases^(^ ^20^ ^)^. T_reg_, characterised by the expression of surface antigens CD4 and CD25 and the transcription factor forkhead box P3 (FOXP3), control inflammation by suppressing the function of T_eff_. T_reg_ are thought to maintain immune system homeostasis and tolerance to self-antigens and non-self-antigens^(^ ^21^ ^–^ ^23^ ^)^.

In the present study, we investigated the role of lactose as a potential inhibitor of human T_reg_-mediated immune regulation in Th1 and Th17 immune responses to evaluate the possible effects of dietary lactose on immune function in humans.

## Materials and methods

### Isolation of human peripheral blood mononuclear cells and enrichment of T cells

**Fig. 1:**
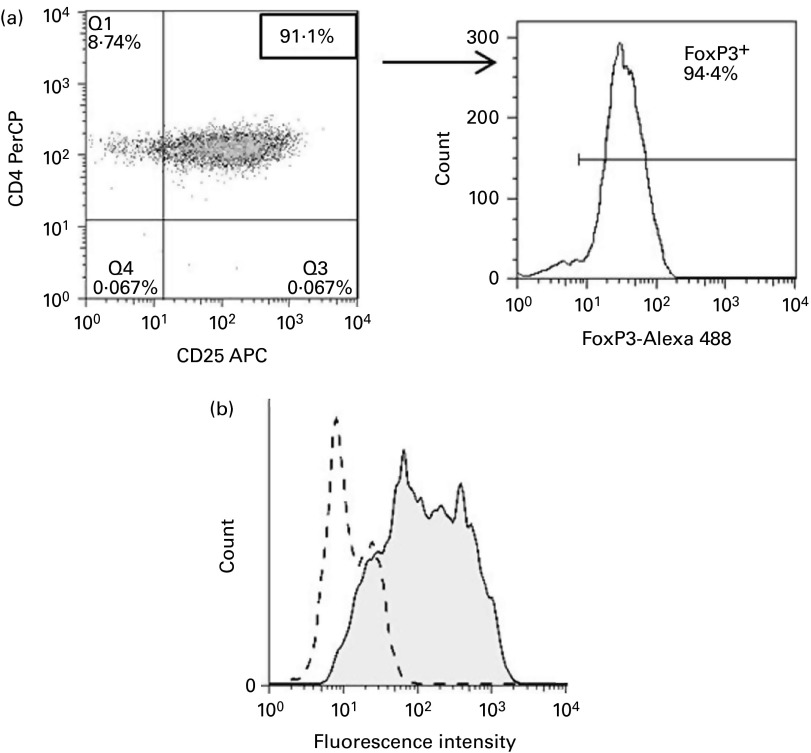
Characterisation of human regulatory T cells (T_reg_) enriched from peripheral blood mononuclear cells using immunomagnetic beads. (a) A fluorescence-activated cell sorting-based phenotype analysis of enriched T_reg_ in lymphocyte gate. Typically, over 94 % of gated CD4^+^CD25^+^ cells expressed the transcription factor forkhead box P3 (FOXP3), a marker for T_reg_. (b) High intracellular protein expression of galectin-9 (Gal-9) in stimulated T_reg_ after 6 d of anti-CD3 and anti-CD28 stimulation. 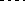, IgG1-phycoerythrin of stimulated T_reg_; 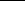, Gal-9-phycoerythrin of stimulated T_reg_. PerCP, peridinin chlorophyll; APC, allophycocyanin.

Peripheral blood mononuclear cells (PBMC) were isolated from twenty healthy donors by Ficoll gradient centrifugation (Ficoll-Paque™ PLUS; GE Healthcare). The collected PBMC were washed three times with PBS (BioWhittaker) and resuspended in Roswell Park Memorial Institute (RPMI) 1640 culture medium (Lonza) supplemented with l-glutamine (Invitrogen), gentamicin (Sigma-Aldrich) and heat-inactivated human AB serum (Innovative Research). Before cell culture, all cell fractions were dyed with Trypan Blue for cell counting and viability assessment. T_reg_ from PBMC populations were enriched using the Regulatory T Cell Isolation Kit II (catalogue no. 130-094-775) according to the manufacturer's recommendation (Miltenyi Biotec). First, PBMC were labelled with a biotinylated antibody cocktail for non-CD4 and CD127 antigens and anti-biotin microbeads, and then the labelled cells were separated magnetically in an LD column (Miltenyi Biotec). Cells passing through the column comprised a pre-enriched CD4^+^CD127^−^ cell population, which was further enriched for T_reg_ by direct magnetic labelling of the surface antigen CD25. CD4^+^CD25^+^CD127^−^ cells were then separated on a magnetic MS column (Miltenyi Biotec). The flow-through fraction of CD4^+^CD127^−^ Th cells that was depleted of CD25^+^ T_reg_ was used as T_eff_. Magnetic separation was performed once for each enriched cell population. The viability of enriched T_reg_ was >89 % and that of enriched T_eff_ was >83 %. The purity of T_reg_ and T_eff_ was assessed by flow cytometry after magnetic separation. Typically, over 94 % of gated CD4^+^CD25^+^ cells, representing T_reg_, expressed the transcription factor FOXP3 (Fig. 1(a)). The CD4^+^CD25^−^CD127^−^ cell population comprising >83 % of CD4^+^ cells was used as T_eff_^(^ ^24^ ^,^ ^25^ ^)^. The present study was conducted according to the guidelines laid down in the Declaration of Helsinki, and all procedures involving human subjects were approved by the ethics committee of the Helsinki University Central Hospital. Written informed consent was obtained from all subjects.

### Cell culture

Enriched T_eff_ and T_reg_ were cultivated in ninety-six-well plates (Thermo Scientific) in CO_2_ incubators at 37°C. The culture medium consisted of RPMI 1640 (Invitrogen) supplemented with human heat-inactivated and sterile-filtered 5 % AB serum, 2 mm-l-glutamine (Invitrogen) and 25 μg/ml gentamicin (Sigma-Aldrich). Before experimentation, the kinetics of Gal-9 expression in stimulated T_reg_ obtained from two healthy individuals was studied. Enriched T_reg_ were stimulated with anti-CD3 and anti-CD28 for 6 d, and the gene expression of *Gal-9* was analysed at 24 h intervals. The peak transcription of *Gal-9* occurred after 6 d of polyclonal stimulation of T_reg_ (data not shown). Based on these results, T_reg_ were pre-stimulated for 4 d before the addition of lactose to the co-cultures to modulate up-regulated endogenous Gal-9 expression. The expression of Gal-9 protein was analysed by flow cytometry in stimulated T_reg_ after 6 d of stimulation.

To study the effects of lactose on the function of T_reg_, first T_reg_ and T_eff_ were stimulated with 5 μg/ml plate-bound anti-CD3 (BD Biosciences) and soluble 5 μg/ml anti-CD28 (BD Biosciences) in separate culture wells for 4 d. Then, T_reg_ were transferred into a co-culture with T_eff_ at a cell ratio of 1:5 (15 000 T_reg_:75 000 T_eff_ in 100 μl volume per well), and 30 mm-lactose (Fluka^®^ Analytical), 30 mm-sucrose (Fisher Scientific) or culture medium without added sugars was added to the cultures. As controls, the T_eff_ were cultured alone or with only lactose. Cell-culture supernatants were collected 3 d after the addition of sugars and stored as such at − 70°C, and cultured cells were collected and lysed in RLT buffer (Qiagen) and stored at − 70°C.

### ELISA

A modified ELISA was used for measuring interferon-γ (IFN-γ) secretion in cell-culture supernatants. Enhanced binding plates (Thermo Scientific) were coated with human IFN-γ capture antibody (Thermo Fisher Scientific) in a binding buffer (0·1 m-Na_2_HPO_4_) and incubated overnight at +4°C. Blocking was performed using 1 % bovine serum albumin in PBS. The plates were washed with 0·05 % Tween in PBS. IFN-γ in undiluted culture supernatant samples was detected using biotinylated secondary IFN-γ antibody (Thermo Fisher Scientific) and biotin-specific streptavidin–alkaline phosphatase (Invitrogen) with *p*-nitrophenylphosphate (Sigma-Aldrich) for colour formation and intensity readings at 405 nm. Recombinant human IFN-γ (R&D Systems) at different dilutions was used for constructing a standard curve for calculation of the concentration of secreted IFN-γ in the samples. Secreted IL-17A in cell-culture supernatants was detected using the Human IL-17 DuoSet ELISA Kit (catalogue no. DY317) according to the manufacturer's instructions (R&D Systems). To prevent inter-assay variation, the supernatant samples from one experiment including different treatments were always analysed in the same assay, i.e. on the same ELISA plate. The detection limit was determined as the lowest standard dilution in the analysis (0·78 ng/ml for IFN-γ and 15·6 pg/ml for IL-17A).

### Quantitative RT-PCR

Total RNA was extracted from pelleted and lysed cultured cells using the RNeasy Mini Kit (Qiagen) with on-column DNase I treatment. High-Capacity cDNA Reverse Transcription Kit (Applied Biosystems) was used for reverse transcription. Real-time detection of target gene complementary DNA amplification was performed using TaqMan Gene Expression Assays (Applied Biosystems) for IFN-γ (Hs00174143_m1) and StepOnePlus instrument (Applied Biosystems) for IL-17A (Hs00174383_m1). RN18S1 (Hs03928985_g1) was used as an endogenous reference gene to calculate comparative/Δ cycle threshold 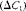 values for IFN-γ complementary DNA and IL-17 complementary DNA amplification. The 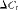 values of target gene amplification were compared with those of an in-house calibrator sample for relative values of gene expression.

### Flow cytometry

The purity of enriched T_eff_ and T_reg_ was verified by staining with anti-human CD3-phycoerythrin, CD4-peridinin chlorophyll, CD8-fluorescein isothiocyanate, CD14-allophycocyanin and CD25-allophycocyanin (Becton Dickinson) and with appropriate IgG1 isotype control (Becton Dickinson) and incubating at room temperature for 20 min. Intranuclear staining for FOXP3 was performed with anti-human FoxP3-Alexa 488 (BioLegend) and isotype control IgG1 (BioLegend) after fixation and permeabilisation using the FoxP3 Fix/Perm Kit (BioLegend). Stimulated cells were incubated with GolgiStop (BD Biosciences) for 4 h and stained with anti-human CD4 and anti-human TIM-3-allophycocyanin (eBioscience) before intracellular staining with anti-human IFN-γ-fluorescein isothiocyanate (BD Pharmingen) and anti-human IL-17A-phycoerythrin (eBioscience), which was performed using the BD Cytofix/Cytoperm Fixation/Permeabilization Kit (BD Biosciences). Gal-9 in stimulated T_reg_ was stained intracellularly with human anti-Gal9 (BioLegend) and IgG1κ (BioLegend) for isotype control using the BD Cytofix/Cytoperm Fixation/Permeabilization Kit (BD Biosciences). For analysis of fluorescence intensity, cells were collected and resuspended in 300 μl of 0·5 % bovine serum albumin in PBS and detected using a FACSCalibur flow cytometer and CellQuest Pro software (Becton Dickinson). Results were analysed using FlowJo 7.6 software (Tree Star, Inc.).

### Statistical analysis

The normality of quantitative RT-PCR and ELISA data was tested, and the data were found to not follow Gaussian distribution. Statistical differences between multiple groups were calculated using the paired non-parametric Friedman test. Statistical differences between two data groups were analysed using the paired non-parametric Wilcoxon test. Data analysis was carried out using GraphPad Prism 6 software (GraphPad Software, Inc.). Statistical significance was set at *P*< 0·05.

## Results

### Human regulatory T cells produce galectin-9 after stimulation

The kinetics of Gal-9 expression in stimulated T_reg_ collected from two different individuals was studied to determine the optimal time to assess the effects of lactose on Gal-9-mediated suppression. Enriched T_reg_ were stimulated with anti-CD3 and anti-CD28 for 6 d, and the gene expression of *Gal-9* was analysed at 24 h intervals. The peak transcription of *Gal-9* occurred after 6 d of polyclonal stimulation of T_reg_ (data not shown). Intracellular Gal-9 production was also detected in enriched human T_reg_, i.e. CD4^+^CD25^+^CD127^−^ after stimulation with anti-CD3 and anti-CD28 for 6 d (Fig. 1).

### Lactose inhibits regulatory T-cell-mediated down-regulation of pro-inflammatory cytokine production

**Fig. 2:**
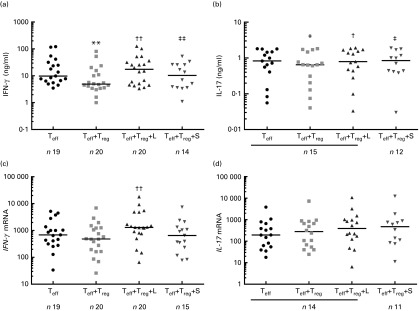
Modulation of the functions of effector T cells (T_eff_) by regulatory T cells (T_reg_) and lactose (L) in healthy study subjects. T_eff_ were cultured as such and in co-cultures with T_reg_ (T_eff_+T_reg_) and with T_reg_+L (T_eff_+T_reg_+L) or sucrose (T_eff_+T_reg_+S). T_reg_-mediated down-regulation of interferon-γ (IFN-γ) (a) and IL-17 (b) secretion was inhibited by lactose. Lactose increased the transcription of *IFN-γ* (c), but not of *IL-17* (d), in T_eff_ co-cultured with T_reg_. Data are represented as minimum to maximum with the median represented by a horizontal line. Cytokine levels in cell-culture supernatants were assessed with ELISA and relative gene expression of the cells was assessed with quantitative RT-PCR. *P* values were calculated using the Wilcoxon signed-rank test for paired samples. Median value was significantly different from that obtained for T_eff_: * *P*= 0·04; ** *P*= 0·003. Median value was significantly different from that obtained for T_eff_+T_reg_: † *P*= 0·005; †† *P*< 0·0001. Median value was significantly different from that obtained for T_eff_+T_reg_+L: ‡ *P*= 0·01; ‡‡ *P*= 0·0002.

To measure the effects of lactose on T_reg_-mediated down-regulation of T_eff_ pro-inflammatory IFN-γ and IL-17 cytokine production, T_eff_ were cultured as such and in co-cultures with T_reg_. In the presence of T_reg_, there was a decrease in the levels of IFN-γ and IL-17 secreted by T_eff_ from a median of 8·8 to 3·9 ng/ml for IFN-γ (Fig. 2(a); *P*= 0·003) and from 0·83 to 0·64 ng/ml for IL-17 (Fig. 2(b); *P*= 0·04). T_reg_-mediated suppression was inhibited when lactose was added to the cell culture, which led to an elevation in the levels of secreted IFN-γ (Fig. 2(a); median 16·4 *v*. 3·9 ng/ml, *P*< 0·0001) and IL-17 (Fig. 2(b); median 0·74 *v*. 0·64 ng/ml, *P*= 0·005). No inhibitory effect of T_reg_ could be observed on the transcription of *IFN-γ* or *IL-17* (Fig. 2(c) and (d)); however, there was an increase in the relative levels of *IFN-γ* transcripts from a median of 484 to 1294 when lactose was added to the co-culture (Fig. 2(c); *P*< 0·0001). No changes were observed in the levels of IFN-γ secreted by stimulated T_eff_ cultured with lactose when compared with those secreted by stimulated T_eff_ cultured without lactose (median IFN-γ values for T_eff_= 38·2 ng/ml, range = 14·86–62·6 ng/ml, and for T_eff_+lactose = 41·4 ng/ml, range = 3·1–64·5 ng/ml, *n* 7, *P*= 0·69).

**Fig. 3:**
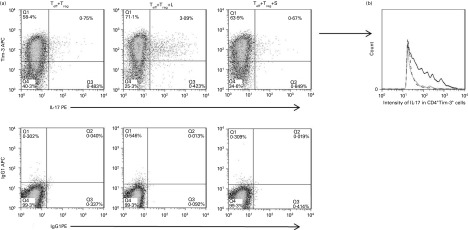
Response exhibited by some individuals to lactose (L) through up-regulation of IL-17 production in CD4^+^TIM-3^+^ cells. The percentage (a) and fluorescence intensity (b) of IL-17 in CD4^+^TIM-3^+^ cells were increased in the presence of lactose; data for a representative case of one individual in whom lactose induced an increase in the IL-17 response of CD4^+^TIM-3^+^ cells. T_eff_, effector T cells; T_reg_, regulatory T cells; S, sucrose; TIM-3, T-cell Ig and mucin domain-3; APC, allophycocyanin; PE, phycoerythrin. 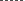, T_eff_+T_reg_; 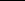, T_eff_+T_reg_+L; 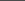, T_eff_+T_reg_+S.

No changes could be observed in the percentage or fluorescence intensity of IFN-γ-producing CD4^+^TIM-3^+^ cells when cultured with T_reg_ with or without lactose (*n* 10). However, in three of the nine blood donors, lactose, but not sucrose, increased the percentage of IL-17-producing CD4^+^TIM-3^+^ cells and the intensity of IL-17 in CD4^+^TIM-3^+^ cells (data of one representative individual shown in Fig. 3).

## Discussion

Our interest in dietary lactose as an immunomodulatory molecule results from studies showing that the Tim-3/Gal-9 pathway is crucial for regulating T-cell responses both *in vitro* and *in vivo* and can be blocked with lactose. In the present study, we showed in a remarkable number of healthy individuals that human T_reg_-mediated down-regulation of Th1 and Th17 immune responses is specifically inhibited by lactose, as evidenced by an increased expression of IFN-γ and IL-17 *in vitro*. The suppressive effect of T_reg_ on IFN-γ expression at both the transcriptional and protein levels was blocked by lactose, which emphasises the importance of Gal-9 as a mediator of immune regulation expressed by T_reg_ and the role of lactose as a potent immunomodulator. When T_eff_ were stimulated with lactose, no changes were observed in the secretion of IFN-γ. This indicates that the effects of lactose were mediated by the inhibition of T_reg_-mediated suppression and not by direct effects on T_eff_. We also provide preliminary evidence that lactose may increase IL-17 responses in CD4^+^TIM-3^+^ cells in some individuals. The results of the present study are in agreement with a recent report showing that human T_reg_ express Gal-9 and that lactose can block Gal-9-mediated suppression of HIV-specific CD8^+^ cells in humans^(^ ^26^ ^)^. In addition, it has been demonstrated that human T-cell-derived Gal-9 is a regulator of Th17/T_reg_ development^(^ ^27^ ^)^.

Human breast milk, containing 7 % lactose, provides infants with nutrients and immunoprotection, in the form of maternal antibodies, antimicrobial peptides, immune cells and cytokines^(^ ^28^ ^,^ ^29^ ^)^. Neonates are exposed to enormous amounts of new microbes, non-pathogens and pathogens and are particularly susceptible to infection. The adaptive immune system of a neonate is immature and Th2-biased and the neonatal immunity relies strongly on innate immunity mechanisms^(^ ^30^ ^,^ ^31^ ^)^. Cederlund *et al.* ^(^ ^32^ ^)^ are the first to show that breast milk lactose exhibits immunomodulatory properties by inducing the transcription of the cathelicidin antimicrobial peptide (*CAMP*), gene encoding the antimicrobial protein LL-37 in colonic epithelial cells and in cells of the innate immune system. We propose that breast milk lactose could have beneficial effects on immunity during infancy by indirectly enhancing the IFN-γ and IL-17 responses of T_eff_. Breast milk lactose could thus be an important mediator of immunoprotection against mucosal pathogens, as shown in an animal model by Sehrawat *et al.* ^(^ ^16^ ^)^. It has been demonstrated that disaccharides such as lactulose, which is used for the assessment of small-intestinal permeability, cross the intestinal barrier in infants and also in individuals with increased intestinal permeability^(^ ^33^ ^)^. In addition, milk oligosaccharides from dietary sources have been shown to interact with cells of the innate immune system in the lamina propria and to promote intestinal inflammation through interaction of sialyl(α2,3)lactose and Toll-like receptor 4 in a mouse model of colitis^(^ ^34^ ^)^. This provides evidence that food-derived oligosaccharides might play a role in the regulation of mucosal immunity in the intestine.

Given that oligosaccharides reach lamina propria, it is plausible that in individuals susceptible to chronic inflammatory diseases, dietary lactose could induce harmful inflammatory responses by disrupting T_reg_-mediated regulation as shown in the present study. The incidence of autoimmune diseases, chronic inflammatory disorders and allergy has increased during the last few decades, especially in Western societies, and cannot be explained by changes in genetic predisposition. Versatile environmental factors are thought to play a key role in these immune-mediated disorders as reviewed by Mohan^(^ ^35^ ^)^ and Smyk *et al.* ^(^ ^36^ ^)^. Uncontrolled Th1 and Th17 immune responses and the inability of T_reg_ to down-regulate immune responses have been implicated in the pathogenesis of many human immune-mediated diseases^(^ ^37^ ^)^. Moreover, Gal-9 has been shown to inhibit IgE–antigen complex formation and mast cell degranulation and alleviate allergic status in mice. The anti-allergic effect of Gal-9 has been found to be completely inhibited by lactose^(^ ^12^ ^)^. As mast cells are also located in the intestinal lamina propria with implications in gastrointestinal disease^(^ ^38^ ^)^, the role of dietary lactose in the exacerbation of allergic inflammation in individuals with food allergy should be considered. In populations with a high proportion of lactose-tolerant individuals, due to the high frequency of lactase gene mutation contributing to the persistent intestinal lactase production during adulthood, lactose intake from the diet is relatively eminent^(^ ^4^ ^,^ ^39^ ^,^ ^40^ ^)^. Interestingly, the incidence of some immune-mediated diseases is high among these populations^(^ ^41^ ^–^ ^43^ ^)^. It is also possible that the intestinal problems associated with genetic lactose intolerance may not always be caused by lactose-related osmotic changes, but could be of immunological origin.

Taken together, lactose has strong immune-modulating properties, which we have demonstrated in the present study *in vitro* in human subjects and which have been shown earlier both *in vitro* and *in vivo* in mice. Physiological relevance of our preliminary results and effects of dietary lactose on the human gut immune system and health need to be studied further.
